# Major ampullate silk gland transcriptomes and fibre proteomes of the golden orb-weavers, *Nephila plumipes* and *Nephila pilipes* (Araneae: Nephilidae)

**DOI:** 10.1371/journal.pone.0204243

**Published:** 2018-10-17

**Authors:** Alessandra D. Whaite, Tianfang Wang, Joanne Macdonald, Scott F. Cummins

**Affiliations:** 1 GeneCology Research Centre and School of Science and Engineering, University of the Sunshine Coast, Sippy Downs, Queensland, Australia; 2 Division of Experimental Therapeutics, Columbia University, New York City, New York, United States of America; Instituto Butantan, BRAZIL

## Abstract

Natural spider silk is one of the world’s toughest proteinaceous materials, yet a truly biomimetic spider silk is elusive even after several decades of intense focus. In this study, Next-Generation Sequencing was utilised to produce transcriptomes of the major ampullate gland of two Australian golden orb-weavers, *Nephila plumipes* and *Nephila pilipes*, in order to identify highly expressed predicted proteins that may co-factor in the construction of the final polymer. Furthermore, proteomics was performed by liquid chromatography tandem-mass spectroscopy to analyse the natural solid silk fibre of each species to confirm highly expressed predicted proteins within the silk gland are present in the final silk product. We assembled the silk gland transcriptomes of *N*. *plumipes* and *N*. *pilipes* into 69,812 and 70,123 contigs, respectively. Gene expression analysis revealed that silk gene sequences were among the most highly expressed and we were able to procure silk sequences from both species in excess of 1,300 amino acids. However, some of the genes with the highest expression values were not able to be identified from our proteomic analysis. Proteome analysis of “reeled” silk fibres of *N*. *plumipes* and *N*. *pilipes* revealed 29 and 18 proteins, respectively, most of which were identified as silk fibre proteins. This study is the first silk gland specific transcriptome and proteome analysis for these species and will assist in the future development of a biomimetic spider silk.

## Introduction

Spider silk is an outstanding proteinaceous fibre that outperforms other natural and synthetic fibres in tensile strength analyses. In addition to fibre strength, spider silks can also be tough, lightweight, highly extensible/flexible, biodegradable and stable across a broad temperature range [1]. Studies have also demonstrated the biocompatibility of spider silk: spider silk can be implanted into living tissue without eliciting an immune response [2, 3]. The potential of all these qualities present in one fibre type is unique and has led to intensive research interest in the molecular structure of the various silks spiders produce.

Orb-weaving spiders have up to seven different glands associated with producing silks and glues used for web architecture [major ampullate gland (MA), minor ampullate (Mi), flagelliform (Flag), aggregate (Ag), and pyriform (Py) glands], protection of eggs [tubuliform glands (Tu)], and prey wrapping [aciniform glands (Ac)] [4]. The molecular structure underpinning each of these silks and glues is the key to understanding the differing inherent mechanical properties of each type [5]. The major constituents in each silk gland are spider silk fibroins, called spidroins (Sp). While spidroins were named according to the gland from which they were primarily identified (for example the major ampullate spidroin (MaSp) from the MA gland), spidroins are not exclusively produced in their namesake gland, and proteomes have revealed silk to contain hundreds of proteins other than those derived from the spidroin gene family [6–11].

In the silk glands, silk proteins are secreted and then stored in the sac-like section of the gland in a water-soluble “molten fibril” or “aqua-melt” state, before processing occurs between the gland, duct and spinneret to produce the insoluble fibre we recognise within webs [12, 13]. Spidroin sequences are very large (approx. >10 kb) and code for a sequence that includes many repetitions of several repeat motifs, and is flanked by non-repetitive, highly conserved N- and C- termini [5,14–16]. Typical repeat motifs in MA protein residues include stretches of poly-alanine (poly-A) and repeat glycine (G) motifs, such as GGX (where X could be A, glutamine, or tyrosine) [14]. An alternate MA spidroin contains stretches of poly-A and contains GPGGX motifs, where proline (P) is believed to contribute to the greater extensibility of this spidroin [10, 17].

Beyond spidroins, much remains to be understood about the tertiary and quaternary interactions of the various proteins and glycoproteins from storage in the soluble phase within the gland, to assembly via processing steps within the duct, and finally, the solid phase fibre as it leaves the spinnerets [18–20]. Synthetic silks have not obtained the level of mechanical performance of their natural counterparts [21–23]. The factors inhibiting successful biomimicry include the inability to mimic the natural production process in current expression systems, including the inability to express full-length recombinant spidroins, and, possibly, the lack of concurrent expression of a set of spidroins and other molecular components found within a silk thread. The non-repetitive N- and C- termini of ampullate silks have been found to play a role in fibre storage and assembly [24]. Further, the N- and C-termini are also thought to aid assembly of the secondary structures of the repeat regions within the processing duct. Indeed, thus far, the best example of a biomimetic spidroin has included these important highly conserved regions flanking a short repeat region [25].

The total primary MaSp sequence has been identified within 3 species; *Latrodectus hesperus*, also known as the Western Black Widow spider, the golden orb-weaving banana spider, *Nephila clavipes*, and *Argiope bruennichi*, the Wasp Spider [14, 26, 27]. Of more than 44,000 spider species, entire spider genomes have only been described for 6 species: *L*. *hesperus* (GenBank: JJRX00000000.1); the brown recluse, *Loxosceles reclusa* (JJRW000000000.1); the common house spider, *Parasteatoda tepidariorum* (AOMJ00000000.2); the African social velvet spider, *Stegodyphus mimosarum* (AZAQ00000000.1); the Brazilian white-knee tarantula, *Acanthoscurria geniculata* (GCA_000661875.1); and, most recently, *N*. *clavipes* (MWRG00000000.1)[27–29]. In combination with their genome assembly for *N*. *clavipes*, Babb et al. characterised spidroin expression within the distinct silk glands [27]. Significant transcriptome coverage has also been achieved by Prosdocimi and colleagues in their infraorder comparative study of expressed RNAs of the spinning glands [30]; by Clark et al., based on multi-tissue de novo transcriptome assemblies of closely related cob-weavers [31–33]; and Correa-Garhwal et al. examined silk expression in male spiders of the family Theridiidae [34].

In Queensland, Australia, *Nephila plumipes* (Latreille, 1804) and *Nephila pilipes* (Fabricius, 1793) are commonly encountered golden orb-weaving species [35]. Studies on *N*. *plumipes* have reported on the mechanical properties of their silk, the relationship between protein secondary structure and primary amino acid sequence, populations in the urban environment, and copulation behaviour, however, no transcriptome data is available on the MA gland of this species, and only a handful of silk sequences are available for *N*. *pilipes* in online databases [36–42]. In this study, we report on a silk-gland specific transcriptome analysis for these golden orb-weaving species, *N*. *plumipes* and *N*. *pilipes*. Furthermore, proteomic analysis of silk fibres from these species was undertaken to compare predominant proteins within the silk fibre to predominant proteins expressed within the MA gland transcriptome. This study found that the silk gland transcriptome of *N*. *plumipes* and *N*. *pilipes* could be assembled into contiguous sequences, and proteome analysis of “reeled” silk fibres could confirm, and be used to mine for, spidroins within the transcriptomes. Novel proteins, which may be important constituents in the structure of spider silk, were also discovered in the silk proteome.

## Methods

### Animals and preparation of RNA

Golden orb spiders of the genus *Nephila plumipes* and *Nephila pilipes* were collected from the Sunshine Coast (26°41'43.1"S 153°05'56.7"E) and the Cooloola Coast (25°54'02.8"S 153°05'25.6"E) regions of Queensland, Australia, between the months of March and June 2013, and February and April 2016. Specimens were dissected immediately after sacrifice and each major ampullate gland were removed and either stored in RNAlater (Invitrogen) or immediately frozen in liquid nitrogen.

RNA was isolated from a pair of MA glands from an individual spider from each species using two different methods; the PicoPure RNA Isolation kit (Arcturus) and TRIzol Reagent (Invitrogen), according to manufacturer’s instructions, with the following changes:

For the PicoPure RNA extraction, an additional RNA purification step was performed using LiCl precipitation. Total RNA (30 μL) was divided into two aliquots for each species, and volume restored to 30 μL with 15 μL of RNase-free water so that samples were in duplicate. All RNA samples were incubated in 2.5 M LiCl (9.4 μL of 8 M LiCl) and 2.5 volumes (98.5 μL) of 100% EtOH for 1.5 h at -20°C. After incubation, the RNA was pelleted at 20,000 x *g* for 20 min at 4°C and the supernatant discarded. The pellet was washed with 70% EtOH and spun at 20,000 x *g* for 10 min at 4°C. The pellet was dried for 10 min at 37°C. The RNA concentration of a second aliquot from each species was estimated spectrophotometrically (NanoDrop 2000) after rehydration with 30 μL RNase-free water to ensure OD_260_/OD_280_ range was between 1.8 and 2.0. Dried samples of the first aliquot were stored at -80°C until shipment to BGI (China) for *de novo* RNA sequencing and bioinformatics.

For the TRIzol RNA extraction, all centrifugation steps were performed at 12,000 rpm at 4°C. RNA concentration and purity was estimated spectrophotometrically (NanoDrop 2000) to ensure OD_260_/OD_280_ range was between 1.8 and 2.0. Resuspended RNA was stored at -80°C until shipment to AGRF (Australia) for *de novo* RNA sequencing.

### Next-generation sequencing, assembly and annotation

Total RNA from paired MA glands from individuals of both spider species were provided to the BGI for *de novo* RNA sequencing and bioinformatics using Illumina HiSeq 2000, and to the AGRF for library construction and paired-end sequencing using an Illumina HiSeq 2500 platform. Raw sequences were assembled into contigs using the Genomic CLC Workbench 9 software (default settings). Protein-coding regions were determined using the open reading frame (ORF) predictor [http://bioinformatics.ysu.edu/tools/OrfPredictor.html]. Blast2GO was utilised for functional annotation of protein-coding regions against the NCBI nr database [43]. Relative expression of genes in the transcriptome was determined based on reads per kilobase of transcript per million mapped reads (RPKM) values, utilizing the *de novo* RNA-seq CLC Genomic Workbench 9 software: transcripts per kilobase million mapped reads (TPM) are also reported.

Spidroin sequences of the genus *Nephila* were obtained from NCBI, compiled and used in a BLASTp search to identify homologous proteins derived from the *N*. *plumipes* and *N*. *pilipes* gland transcriptomes. Matches were manually assessed to determine conservation. Further, a “spidroin-like” database was created by examining the six translated nucleotide reading frames for the following spidroin-like amino acid motifs: AAAAA, GGYGG, GYGPG, GQQGP, and GAGAGG. Finally, CD-search [https://www.ncbi.nlm.nih.gov/Structure/bwrpsb/bwrpsb.cgi?] was utilised to identify specific hits to spidroin protein domains and superfamilies.

### Spider silk preparation for proteomics

Spider silk threads were obtained by hand-reeling silk straight from live *N*. *plumipes* and *N*. *pilipes* spiders. Proteins were extracted from the silk by homogenisation in 100 μL protein extraction buffer (7 M urea, 2 M thiourea, 4% (w/v) CHAPS, 65 mM DTT), to which 100 μL of 30 mM Tris (pH 7.5) was added. Homogenate was vortexed and pulse-centrifuged several times to mix the dissolution buffer and Tris solution. The homogenate was vortexed for 15 min, pulse centrifuged, and then incubated in a sonicating water bath at ambient temperature for 20 min. Undissolved substances were pelleted by centrifugation at 12,000 x *g* for 8 min, and the supernatant containing the dissolved proteins were collected and stored at -80°C.

### Sodium dodecyl sulphate—polyacrylamide gel electrophoresis (SDS-PAGE) and in-gel trypsin digestion

The spider silk proteins were separated by SDS-PAGE using a 4–20% polyacrylamide gradient gel (Amersham ECL Gel, GE Healthcare Life Sciences) according to manufacturer’s instructions. Samples were prepared 1:1 with 2x SDS sample buffer and incubated at 95°C for 5 min, then cooled to room temperature prior to pulse centrifugation. Samples were run for 1 h at 160 V (45 mA). Separated proteins were visualised by Coomassie Brilliant Blue (Bio-Rad) according to the staining process recommended by GE Healthcare. Upon completion of electrophoresis the proteins were precipitated with fixing solution [400 mL of EtOH, 100 mL of acetic acid, 500 mL of distilled water (DI)] for 30 min, followed by immersion in staining solution (1 tablet of PhastGel Blue-R 350, 400 mL of destaining solution; 250 mL of EtOH, 80 mL of acetic acid, 670 mL DI) for 10 min. The gel was subsequently preserved by immersion in preserving solution (25 mL of (87% v/v) glycerol with DI, 225 mL destaining solution) for 30 min. Protein sizes were estimated using a Pierce Blue Molecular Weight marker (Thermo Scientific).

Excised protein bands were washed in 50 mM NH_4_HCO_3_ at room temperature for 5 min, and then de-stained by incubating gel pieces for 30 min in 50 mM NH_4_HCO_3_ in 30% acetonitrile in a sonication water bath to remove Coomassie Brilliant Blue. Gel pieces were subsequently collected by in-gel trypsin digestion using the method described elsewhere [44]. The samples were reconstituted in 0.1% formic acid and stored at -20°C until mass spectroscopy.

### NanoLC tandem TripleTof MS/MS analyses and protein identification

The spider silk extracts were analysed by LC-MS/MS on a Shimadzu Prominence Nano HPLC (Japan) coupled to a Triple ToF 5600 mass spectrometer (ABSCIEX, Canada) equipped with a nano electrospray ion source. Each extract (7 μL) was injected onto a 50 mm x 300 μm C18 trap column (Agilent Technologies, Australia) at 30 μL/min. The samples were de-salted on the trap column for 5 minutes using 0.1% formic acid (aq) at 30 μL/min. The trap column was then placed in-line with the analytical nano HPLC column, a 150 mm x 75 μm 300SBC18, 3.5 μm (Agilent Technologies, Australia) for mass spectrometry analysis. Linear gradients of 1–40% solvent B over 35 min at 300 nL/minute flow rate, followed by a steeper gradient from 40% to 80% solvent B in 5 min were used for peptide elution. Solvent B was held at 80% for 5 min for washing the column and returned to 1% solvent B for equilibration prior to the next sample injection. Solvent A consisted of 0.1% formic acid (aq) and solvent B contained 90/10 acetonitrile/ 0.1% formic acid (aq). The ionspray voltage was set to 2400V, declustering potential 100V, curtain gas flow 25, nebuliser gas 1 (GS1) 12 and interface heater at 150°C. The mass spectrometer acquired 500 ms full scan TOF-MS data followed by 20 by 50 ms full scan product ion data in an Information Dependant Acquisition mode. Full scan TOF-MS data was acquired over the mass range 350–1800 and for product ion ms/ms 100–1800. Ions observed in the TOF-MS scan exceeding a threshold of 100 counts and a charge state of +2 to +5 were set to trigger the acquisition of product ion, MS/MS spectra of the resultant 20 most intense ions. The data was acquired and processed using Analyst TF 1.5.1 software (AB SCIEX, Concord, Canada).

The LC-MS/MS data were imported to the PEAKS studio (Bioinformatics Solutions Inc., Waterloo, ON, Canada, version 7.0) with the assistance of MS Data Converter (Beta 1.3, http://sciex.com/software-downloads-x2110). The database search included our own *Nephila sp*. transcriptome-derived protein databases, our “spidroin-like” database made by motif-searching the six translated nucleotide reading frames, and non-redundant protein databases (GenBank and UniProt). *De novo* sequencing of peptides, database search and characterising specific PTMs were used to analyse the raw data; false discovery rate (FDR) was set to ≤ 1%, and [-10*log(p)] was calculated accordingly where p is the probability that an observed match is a random event. The PEAKS used the following parameters: (i) precursor ion mass tolerance, 0.1 Da; (ii) fragment ion mass tolerance, 0.1 Da (the error tolerance); (iii) tryptic enzyme specificity with two missed cleavages allowed; (iv) monoisotopic precursor mass and fragment ion mass; (v) a fixed modification of cysteine carbamidomethylation; and (vi) variable modifications including lysine acetylation, deamidation on asparagine and glutamine, oxidation of methionine and conversion of glutamic acid and glutamine to pyroglutamate.

## Results and discussion

**Fig 1:**
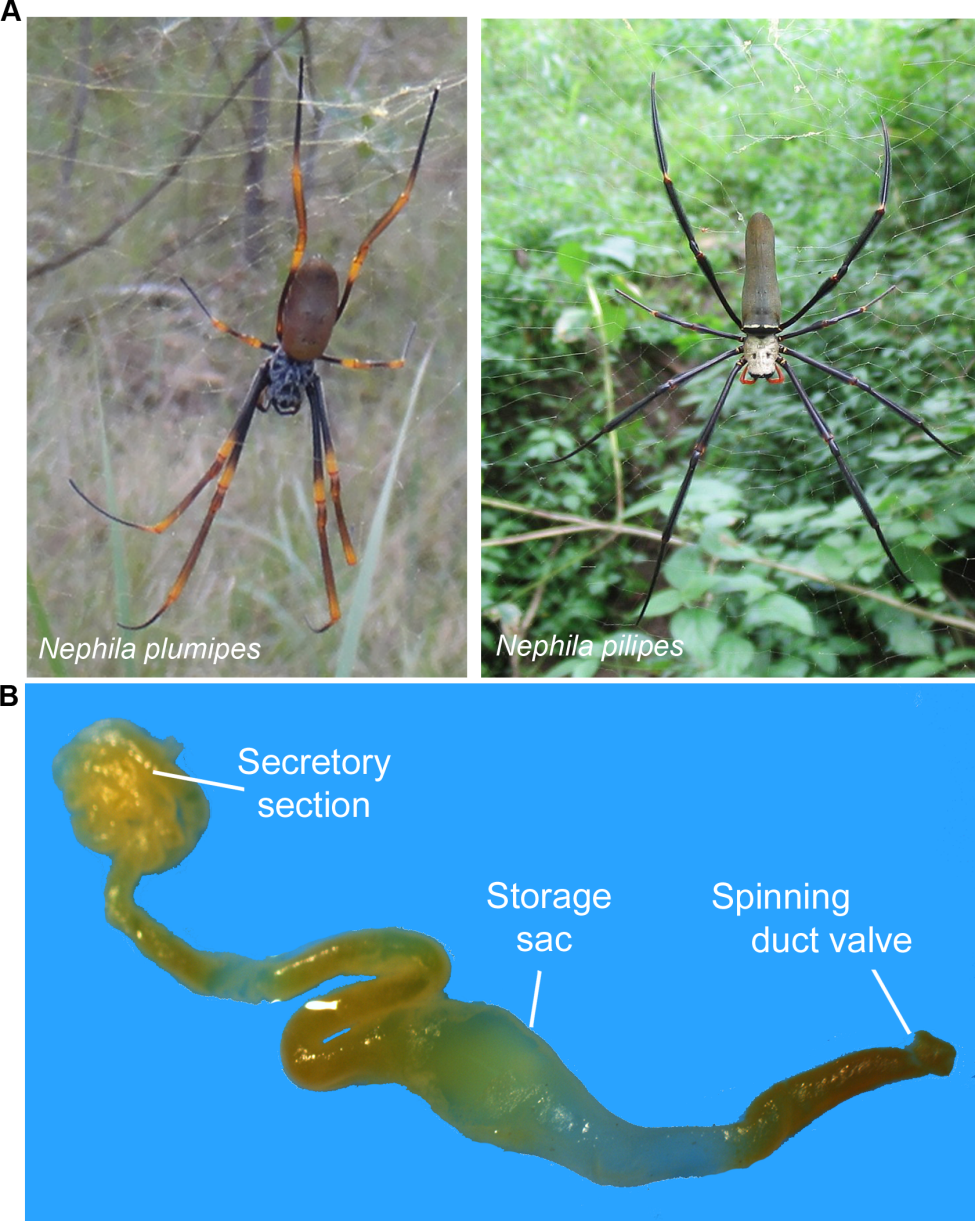
The golden orb-weaving spiders and a representative MA gland. (A) *Nephila plumipes* (photo by Alessandra Whaite) and *Nephila pilipes* (photo by Amos T Fairchild). (B) The major ampullate gland of *Nephila pilipes*, indicating the secretory section and the sac that stores silk proteins.

*Nephila plumipes* and *N*. *pilipes* (Fig 1A) were collected and the MA glands were removed (Fig 1B) for RNA isolation and sequencing. MA gland reference transcriptomes were constructed for each species by combining next-generation sequence (NGS) data from sequencing runs produced in 2013, with data produced in 2016 (GenBank Accession: SRR6747912, SRR6747911). The combined MA gland transcriptomes for *N*. *plumipes* and *N*. *pilipes* produced 42,351,802 and 46,060,170 total paired reads, respectively. Paired reads were assembled into 69,812 contiguous sequences (contigs) for *N*. *plumipes* with an average length of 685 nucleotides, and into 70,123 contigs for *N*. *pilipes* with an average nucleotide length of 672. The data returned from ORF prediction were 67,862 and 67,942 sequences, and Blast2Go annotation allowed for the annotation of approximately 25% and 29% of all transcripts plus identification of 48 and 35 spidroin contigs, for *N*. *plumipes* and *N*. *pilipes*, respectively. The total spidroin count for each species was increased to 73 and 60 spidroins for *N*. *plumipes* and *N*. *pilipes* (Table 1 and 2), upon mining for spidroin sequences identified within each corresponding transcriptome-derived silk proteome (S1 and S2 Tables), by analysing the six translated nucleotide reading frames for spidroin-like repeat motifs, and by BLAST searching unique silk sequences found in the related *N*. *clavipes* genome reported by Babb et al. [27].

**Table 1:** Total spidroins in each *Nephila plumipes* data set (2013 and 2016).

ID	Predicted Spidroin	Length	Domain/ Superfamily	RPKM 2013	RPKM 2016	TPM 2013	TPM 2016
U_9113	AcSp1	97		0.00	0.31	0.00	0.22
U_25766	AcSp1	156		0.00	1.11	0.00	0.82
U_397	AgSp1	319		59.00	149.92	48.18	110.17
U_1689	AgSp1	338		0.00	39.15	0.00	28.77
U_1891	AgSp1	183		2.01	853.75	1.64	627.39
U_25614	AgSp2	48		26.25	3.78	21.43	2.78
U_7370	AgSp-a	523	Spidroin_N superfamily	0.00	8.89	0.00	6.54
U_1394	AgSp-c	1391		0.16	11.22	0.13	8.25
U_85	AgSp-c	761		30.92	413.08	25.25	303.56
U_6753	AgSp-c	127		0.00	143.05	0.00	105.12
U_6110	AgSp-c	182	Spidroin_N superfamily	0.00	7.40	0.00	5.44
U_2628	ECP-1†	198		0.00	4088.26	0.00	3004.33
U_4754	Flag1	226		43.48	2.19	35.51	1.61
U_20913	Flag2	107		0.00	0.48	0.00	0.35
U_18953	MaSp	89		8697.28	85599.66	7102.42	62904.26
U_10053	MaSp	88		41232.70	10475.45	33671.66	7698.05
U_11966	MaSp	99		2879.08	16061.76	2351.13	11803.24
U_17526	MaSp	71		1395.00	11596.29	1139.19	8521.72
U_1088	MaSp	103		4472.41	4888.34	3652.28	3592.28
U_47	MaSp1	57	Spidroin_MaSp	16143.09	6830.23	13182.85	5019.30
U_28059	MaSp1	112	Spidroin_MaSp	106.66	10.81	87.10	7.94
U_56	MaSp1	217	Spidroin_N	6718.57	27671.27	5486.55	20334.67
U_82*	MaSp1	189	Spidroin_N superfamily	6.10	6223.69	4.98	4573.57
U_172	MaSp1	60	Spidroin_MaSp	22158.38	581.88	18095.09	427.61
U_2048	MaSp1	60	Spidroin_N superfamily	0.00	32.84	0.00	24.13
U_4815	MaSp1	41		16.48	81.39	13.45	59.81
U_15285	MaSp1	248	Spidroin_MaSp	169.59	934.33	138.49	686.61
U_49434	MaSp1	130	Spidroin_N superfamily	0.00	0.25	0.00	0.18
U_999*	MaSp1	126		5864.84	50336.66	4789.37	36990.68
U_6045*	MaSp1	96		5861.11	7092.78	4786.33	5212.24
U_9403*	MaSp1	92		52042.69	55197.89	42499.36	40563.04
U_10063*	MaSp1	88		5068.79	30452.03	4139.30	22378.15
U_27155*	MaSp1	73		1217.71	1221.18	994.41	897.41
U_8956*	MaSp1	48		202.72	4187.95	165.55	3077.58
U_25731*	MaSp1	119		39.68	328.95	32.40	241.74
U_33	MaSp1	85	Spidroin_MaSp	39225.86	7270.57	32032.82	5342.90
U_18230	MaSp2	154	Spidroin_N	0.89	6.39	0.73	4.70
U_22058*	MaSp-c	68	Spidroin_N superfamily	15.46	1191.73	12.62	875.76
U_14382*	MaSp-d	125		190.96	9074.23	155.94	6668.34
U_6066*	MaSp-d	114		536.54	46701.72	438.15	34319.50
U_46611	MaSp-f	140		0.00	0.59	0.00	0.43
U_10737	MaSp-f	239	Spidroin_MaSp	13.74	50.18	11.22	36.88
U_1060*	MaSp-g	228		317.41	560.02	259.21	411.54
U_17514*	MaSp-g	68		3181.41	1064.47	2598.02	782.25
U_2249*	MaSp-g	135		2005.01	1405.81	1637.35	1033.08
U_679*	MaSp-g	220		406.52	428.27	331.97	314.72
U_24021	MaSp-h	82	Spidroin_N superfamily	31.96	525.52	26.10	386.19
U_17901	MiSp1	153	Spidroin_MaSp	0.00	2.99	0.00	2.20
U_11571	MiSp1	75	Spidroin_MaSp	224.25	100.05	183.13	73.52
U_12772	MiSp1	253	RP1-2	18.62	22.26	15.21	16.36
U_1636*	MiSp1	336	RP1-2	5.21	58.80	4.26	43.21
U_27015	Sp-5803	111		4.31	0.50	3.52	0.37
U_25915	Sp-74867	118	Spidroin_MaSp	3.79	0.66	3.09	0.48
U_17052	Sp-74867	93	Spidroin_MaSp	9185.22	7487.22	7500.88	5502.10
U_37	Sp-8175	920		1.40	47.08	1.15	34.59
U_6378	Sp-907	277	Spidroin_N superfamily	0.33	32.09	0.27	23.58
U_4542*	Sp-907	69		90.31	588.96	73.75	432.80
U_368*	Sp-907	259		29.90	787.06	24.42	578.38
U_17587*	Sp-907	108		1107.42	2025.01	904.35	1488.11
U_1699*	Sp-907	110		55.68	259.41	45.47	190.63
U_2859*	Sp-907	108		293.37	1928.52	239.57	1417.20
U_15734*	Sp-907	124		65.56	2816.43	53.53	2069.70
U_6377*	Sp-907	102		0.63	25.35	0.52	18.63
U_3*	Sp-907	117		422.86	3432.85	345.32	2522.68
U_319	Sp-907	164		1330.27	12939.36	1086.33	9508.69
U_21571	TuSp	179	Spidroin_MaSp	0.00	8676.94	0.00	6376.38
U_586	TuSp1	241	Spidroin_N	0.00	1486.26	0.00	1092.20
U_897	TuSp1	152	RP1-2	0.00	74973.89	0.00	55095.74
U_3907	TuSp1	183		0.00	43.55	0.00	32.01
U_7486	TuSp1	116	RP1-2	0.00	3.58	0.00	2.63
U_23917	TuSp1	149	Spidroin_N superfamily	0.00	1.32	0.00	0.97
U_60428	TuSp1	119	Spidroin_N superfamily	0.00	0.84	0.00	0.62
U_86	TuSp1	89		0.00	40804.39	0.00	29985.74

RPKM, Reads Per Kilobase of transcript per Million mapped reads

TPM, Transcripts Per Kilobase Million

† ECP-1, egg case protein-1

* Also identified in the corresponding proteome

**Table 2:** Total spidroins in each *Nephila pilipes* data set (2013 and 2016).

ID	Predicted Spidroin	Length	Domain/ Superfamily	RPKM 2013	RPKM 2016	TPM 2013	TPM 2016
I_65000	AcSp	106		0.00	0.16	0.00	0.13
I_55	AcSp1	240	Spidroin_N superfamily	0.00	9455.94	0.00	7450.90
I_29651	AgSp1	143		0.82	1.24	0.55	0.98
I_69410	AgSp1	131		1.04	0.91	0.69	0.72
I_17302	AgSp1	114		5.10	5.89	3.41	4.64
I_4597	AgSp-a	281		0.46	4.64	0.31	3.65
I_17659	AgSp-a	359	Spidroin_N superfamily	0.00	2.94	0.00	2.32
I_1837	AgSp-c	1609	Spidroin_MaSp	18.38	41.31	12.31	32.55
I_31481	AgSp-c	214		0.00	0.69	0.00	0.54
I_13893	AgSp-c	148		2.71	5.22	1.82	4.11
I_1450	AgSp-c	104		10.90	37.31	7.30	29.40
I_67816	AgSp-c	112	Spidroin_N superfamily	1.24	0.47	0.83	0.37
I_18284	Flag	277		23.42	0.72	15.68	0.57
I_14698	Flag1	203	Spidroin_N superfamily	0.00	4.80	0.00	3.78
I_4468	Flag2	245		6.51	93.38	4.36	73.58
I_12595	MaSp	116		17160.99	2766.09	11489.93	2179.57
I_33885	MaSp	110		44532.47	979.27	29816.18	771.62
I_7068	MaSp	79	Spidroin_MaSp	242027.26	87.87	162046.41	69.24
I_228	MaSp1	221	Spidroin_MaSp	689.40	4201.47	461.58	3310.59
I_304*	MaSp1	277		7.87	786.60	5.27	619.81
I_820	MaSp1	173	Spidroin_MaSp	25576.95	2260.04	17124.73	1780.82
I_27560*	MaSp1	105		44.86	3339.47	30.03	2631.37
I_141	MaSp1	306	Spidroin_N superfamily	10.72	3181.16	7.18	2506.63
I_478*	MaSp1	498	Spidroin_N	133.27	362.93	89.23	285.98
I_11111	MaSp1	169	Spidroin_N superfamily	25.36	14.10	16.98	11.11
I_1327*	MaSp1	89		2.63	446.18	1.76	351.57
I_262*	MaSp1	173		2058.66	1616.88	1378.35	1274.04
I_2904*	MaSp1	92		9607.91	2971.56	6432.86	2341.47
I_17778*	MaSp1	79		17.66	1186.59	11.82	934.99
I_2074*	MaSp2	120		138074.73	62.50	92446.26	49.25
I_272*	MaSp2	96	Spidroin_MaSp	238666.86	1906.18	159796.50	1502.00
I_16679	MaSp2	93		60136.69	377.02	40263.79	297.07
I_1340*	MaSp-f	328	Spidroin_MaSp	56.92	344.75	38.11	271.65
I_9111*	MaSp-h	143		53644.90	35.16	35917.29	27.70
I_63355	MaSp-h	104	Spidroin_MaSp	8.03	0.34	5.38	0.26
I_359*	MaSp-h	392		32815.12	2140.32	21970.96	1686.49
I_550	MaSp-h	166		18520.57	5149.23	12400.23	4057.39
I_4156	MiSp	133		10236.26	252.33	6853.56	198.83
I_12574	MiSp	84		0.00	323.96	0.00	255.27
I_10698	MiSp	129		60.56	15064.25	40.55	11870.03
I_20587	MiSp	153		7.17	9866.14	4.80	7774.13
I_4316	MiSp	83		6.03	8829.59	4.04	6957.37
I_26363	MiSp	113		66.04	8262.29	44.22	6510.35
I_3787	MiSp1	66		67.69	13233.55	45.32	10427.51
I_21222	MiSp1	119	Spidroin_MaSp	110.32	7302.47	73.86	5754.06
I_9	MiSp1	136	Spidroin_MaSp	207.34	13762.70	138.82	10844.46
I_7810	MiSp1	95		87.22	9654.94	58.40	7607.71
I_7754	MiSp-a	164		52.36	11827.42	35.05	9319.54
I_8908*	MiSp-d	125		1939.86	211.64	1298.81	166.76
I_61631	Sp-1339	134		0.00	0.50	0.00	0.40
I_52113	Sp-5803	111		19.22	0.84	12.87	0.67
I_20950	Sp-907	147	Spidroin_N superfamily	30.53	19.48	20.44	15.35
I_12762	TuSp1	183	RP1-2	0.00	3.78	0.00	2.98
I_22088	TuSp1	78	Spidroin_MaSp	0.00	14056.52	0.00	11075.98
I_23940	TuSp1	187		0.00	8958.45	0.00	7058.90
I_48	TuSp1	97		0.00	978.98	0.00	771.40
I_1415	TuSp1	112	RP1-2	0.00	6772.23	0.00	5336.25
I_4212	TuSp1	118		0.00	221762.64	0.00	174740.14
I_9940	TuSp1	109	RP1-2 superfamily	0.00	20210.87	0.00	15925.36
I_8400	TuSp1	88		0.00	77285.64	0.00	60898.01

RPKM, Reads Per Kilobase of transcript per Million mapped reads

TPM, Transcripts Per Kilobase Million

* Also identified in the corresponding proteome

A recent paper reporting on the genome and tissue transcriptomes of the golden-orb spider, *N*. *clavipes*, identified 28 spidroins [27]. Our transcriptomes included partial sequences with homology to several of the unique spidroins found in this closely related species, including AgSp-a, AgSp-c, Sp-5803, Sp8175, Sp74867 and, MaSp-c, -d -g and -h (Table 1 and 2). In *N*. *plumipes*, our study uncovered numerous matches to the *N*. *clavipes* spidroin Sp-907, potentially non-overlapping contigs aligning to different regions on the same gene. In *N*. *pilipes*, our study found sequences with homology to MiSp-a and -d, and Sp-1339. Our longest assembled spidroin contigs in both species matched to the *N*. *clavipes* the glue-like aggregate spidroin, AgSp-c. Aggregate spidroins, which form evenly-spaced droplets along flagelliform prey capture threads, have also been characterised in three other species from the family Araneidae, and three species from the family Theridiidae [45, 46], These spidroins vary greatly in length among species and it appears we have recovered a full-length aggregate spidroin (1609 aa) from *N*. *pilipes*. However, aggregate spidroins were not evident in the proteome of either species.

**Fig 2:**
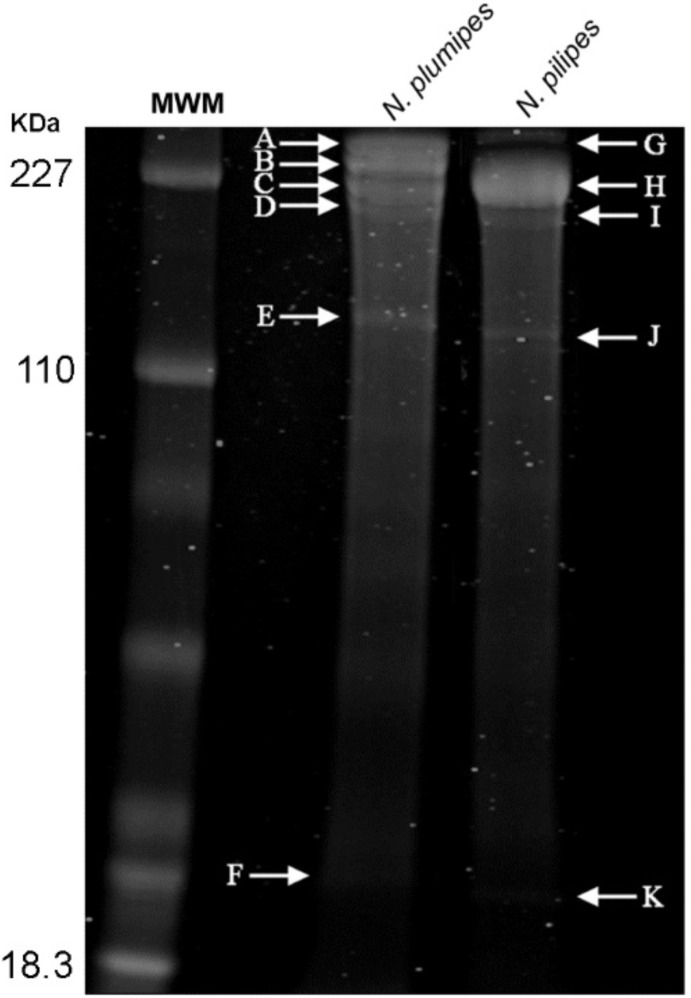
SDS-PAGE and Coomassie stain indicating prominent protein bands that were excised for LC-MS/MS analysis. (A-F) *Nephila plumipes*, (G-K) *Nephila pilipes*. MWM, molecular weight marker.

The *N*. *pilipes* and *N*. *plumipes* silk proteins were obtained after several predominant bands were excised from the Coomassie stained SDS-PAGE gel (Fig 2), followed by trypsin digestion and LC-MS/MS analysis. This analysis identified 29 and 18 proteins for *N*. *plumipes* and *N*. *pilipes* (Table 3 and 4), respectively (≥20.00 -10lgP, ≥ 2 peptide matches). For the silk proteins of *N*. *plumipes* mapped to the transcriptome, 24 were spidroins, mostly MaSp1-like spidroins, MiSp-like and novel *N*. *clavipes* spidroin, Sp-907, confirming the abundance of this spidroin in the transcriptome. Besides spidroins, a cuticle protein and a coiled-coil domain-containing protein were identified in the proteome along with two proteins which could not be identified. Cuticle proteins have been described previously in the MA gland [7, 47]. The coiled-coil domain protein found within the transcriptome and proteome of *N*. *plumipes* is potentially interesting as coiled-coil structures are also found to form in the silk of the Japanese yellow hornet, *Vespa simillima* [48]. *N*. *pilipes* silk proteins accounted for 13 of 18 proteins mapped back to the corresponding transcriptome. Again, most of these spidroins were MaSp-like, one MiSp and the remaining 5 proteins were not able to be annotated at this stage.

**Table 3:** *Nephila plumipes* proteins within the silk matching to the transcriptome.

ID	-10lgP	Cover-age (%)	No. of Peptides	Unique Peptides	PTM	Average Mass	Example Peptides	BLAST Similarity
U_999*	484.05	63	27	15	Amidation; Deamidation (NQ); Pyro-glu from Q	12348	R.GAGAAAAAAGGAGQGGYGGLGSQGAGR.G, G.QGAAAAAAGGAGQGGYGGLGGQGAGR.G	Major ampullate spidroin 1 [Nephila clavipes]
U_1060	403.82	66	17	16	Amidation; Deamidation (NQ); Pyro-glu from Q	19573	R.GPGGYGPGQQGPAQQGPGQQGPGGAGAAAAAGR.G, R.GPGSYGPGQQGPGQQGPR.Q	Major ampullate spidroin protein MaSp-g [Nephila clavipes]
U_434	282.78	23	15	15	Acetylation (K); Deamidation (NQ); Oxidation (M)	80294	R.GGGGGFNVPSGGGGLNIPSGGGR.G, R.DISSSATSASSASAGDAGGIGQGR.N	N/A
U_368	237.58	39	8	7		21576	R.GGDSGAAAAAAAADGGR.G, R.GGDTGAAAAAAAADGGR.G	Spidroin protein Sp-907 [Nephila clavipes]
U_3	172.59	16	7	6		38371	R.GGDSGAAAAAAAADSGR.G, R.GGYGGLGR.G	Spidroin protein Sp-907 [Nephila clavipes]
U_679	141.03	24	4	3	Deamidation (NQ)	18685	R.YGPSGPGSAAAAAAAAGAGSR.G, G.GYGPGQQGPGQQ(+.98)GPGQQG	Major ampullate spidroin protein MaSp-g [Nephila clavipes]
U_1081	102.56	18	4	4	Acetylation (K); Deamidation (NQ); Pyro-glu from Q	16054	K.GYDNDFVR.F, R.Q(-17.03)FDHPYK.R	Hypothetical Protein NCL1 41264 [Nephila clavipes]
U_4542	132.07	29	3	3		5670	L.GGDSGAAAAAAAAADGGR.G, R.GLGGDSGAAAAAAAAADGGR.G	Spidroin protein Sp-907 [Nephila clavipes]
U_82*	90.27	18	3	3	Acetylation (K); Deamidation (NQ); Pyro-glu from Q	18384	K.AFYQTTGTEDSR.F, G.Q(-17.03)VTPWSNAK.L	Major ampullate spidroin 1 variant 1, partial [Nephila clavipes]
U_6066*	159.66	46	2	2	Deamidation (NQ)	10109	R.FGSGGPGGDSAAAAAASGGNGGR.F, R.FGSGGPGGDSAAGAAASGGNGGNGGN(+.98)GGR.F	Major ampullate spidroin protein MaSp-d [Nephila clavipes]
U_6045*	121.31	41	2	2	Deamidation (NQ)	10812	R.AAAAAAGGAGQGGYGSLGSQGAGR.G, G.AGGAAAAAGGAGQ(+.98)GG	Spidroin 1 [Nephila clavipes]
U_22058	116.5	39	2	1	Oxidation (M)	7566	R.TGAFTADQLDDMSTIGDTLK.T, R.TGAFTADQLDDM(+15.99)STIGDTLK.T	Major ampullate spidroin protein MaSp-c [Nephila clavipes]
U_14382*	133.02	35	2	2		7519	R.FGSEGPGGDSAAAAAASGGDGGR.F, R.FGTGGPESDSAAASGGNGGNR.Q	Major ampullate spidroin protein MaSp-d [Nephila clavipes]
U_17587	73.53	23	2	1		8766	G.SSGAAAAAAAADGGIGR.G, S.GGYGGIGR.G	Spidroin protein Sp-907 [Nephila clavipes]
U_1699	55.99	16	2	1		9285	R.GGYGGLGR.G, A.AAAAAAEGGRGGYGGLGR.G	Spidroin protein Sp-907 [Nephila clavipes]
U_1669	27.36	3	2	2		85262	L.DVINSNESR.L, K.QKLSELEVQK.Q	Coiled-coil domain-containing protein [Nephila clavipes]
U_9403	236.31	100	43	36	Acetylation (Protein N-term); Deamidation (NQ); Ethylation; Methyl ester; Acetylation (N-term); 7 more	7117	Q.GAGAAAAAAGGAGQGGYGGLGSQGAGR.G, G.AAAAAAGGAGQGGYGGLGSQGAGR.G	Dragline silk fibroin [Nephila clavipes]
U_2859	151.32	99	30	29	Acetylation (Protein N-term); Deamidation (NQ); Ethylation; Methyl ester; Octanoyl; 6 more	8958	GLGGDSAAAAAAAADGGR.G, G.GDSAAAAAAAADGGR.G	Spidroin protein Sp-907 [Nephila clavipes]
U_8956*	99.15	56	19	19	Carbamidomethylation; Deamidation (NQ); Ethylation; Methyl ester; Octanoyl; 9 more	10187	R.GAGAAAAAAAGGAGQGGYRS(-18.01)EE(+14.02).H, R.GAGAAAAAAAGGAGQGGYRS(-18.01)E(+14.02)E.H	Dragline silk fibroin [Nephila clavipes]
U_2249	57.34	77	14	7	Deamidation (NQ); Ethylation; Methyl ester; Hydroxylation; Dihydroxy; Dehydration	10986	G.GPGGYGPGQQGPGQQGPGQ(+.98)QG.P, P.GGAAAAAAAAGGPGGYGPGQQGP.G	Major ampullate spidroin protein MaSp-g [Nephila clavipes]
U_10063*	98.84	92	12	7	Acetylation (Protein N-term); Deamidation (NQ); Ethylation; Octanoyl	6547	Q.GGYGGQGAGAAAAAGGAGQ.G, Q.GGYGGQGAGAAAAAGGAGQGGQ.G	Major ampullate spidroin-like protein, partial [Nephilengys cruentata]
U_27155	116.09	89	11	8	Acetylation (Protein N-term); Deamidation (NQ)	6231	G.AGAGAAAAAAGGAGQGGYGGLGGQ(+.98)GAGQG.G, G.AGAGAAAAAAGGAGQGGYGGLGGQGAGQ(+.98)G.G	Dragline silk spidroin 1 [Nephila pilipes]
U_17514	68.91	88	11	3	Acetylation (Protein N-term); Deamidation (NQ); Dehydration	5594	Q.Q(+42.01)(+.98)GPSGPGGAAAAAAAA.G, A.GPGGYGPGQQGPGQQGPGQ(+.98)QG.P	Major ampullate spidroin protein MaSp-g [Nephila clavipes]
U_15734	128.72	36	4	3		10272	R.GGDSGAAAAAAAADGGR.G, G.DSGAAAAAAAADGGR.G	Spidroin protein Sp-907 [Nephila clavipes]
U_29163	22.58	12	3	3		39198	G.GAGGGRGGGAGGNYPPQPYN.F, Q.VSIVVAALV.G	Cuticle protein 10.9 [Nephila clavipes]
U_1636	22.56	24	3	3	Deamidation (NQ)	29130	A.AAGGAAGYGRGAGAGAGAAAG.A, S.GAGGGAVAGAGAAAGAV.S	Chain A, 3D structure of RP domain of MiSp
U_6377	46.76	30	2	1		9218	R.GGYGGLGR.G, R.IGYGPGGVSGAAAVAAAADSGKG.S	Spidroin protein Sp-907 [Nephila clavipes]
U_97	23.18	1	2	2	Deamidation (NQ)	361672	V.DASVPGGRHK.S, C.RDISLQ(+.98)NVQK.M	N/A—short sequence
U_25731	20.94	19	2	1		9468	H.GGLGGQGAAAAAAGGAGQGGLGG.L, G.QGAAAAAAGGAGQGGLGG.L	Dragline silk fibroin [Nephila clavipes]

Proteins from the corresponding transcriptome with 2 or more peptide matches were BLAST annotated (E-value cut-off 10^−3^). Example matching peptides are shown (full list, see S3 Table). PTM, posttranslational modifications.

**Table 4:** *Nephila pilipes* proteins within the silk matching to the transcriptome.

ID	-10lgP	Cover-age (%)	No. of Peptides	Unique Peptides	PTM	Average Mass	Example Peptides	BLAST Similarity
I_478	288.93	25	8	8	Deamidation (NQ); Oxidation (M); Pyro-glu from Q	42362	R.QGGQGAGAATAAASGAGQGGYGR.Q, R.QGGQGAGAAAAGAGGAGR.G	Major ampullate spidroin 1 variant 3 [Nephila clavipes]
I_304	261.74	21	2	2		22373	R.NAAVAAAAAGGLGGYGLGGQGSGQR.S, R.PSGAGGQGAQAPGGYGTGSGSTIVITAGGQR.G	Spidroin 1 [Nephila clavipes]
I_33*	233.73	35	8	8	Acetylation (K); Deamidation (NQ); Oxidation (M)	24343	K.DAGGVM(+15.99)QGALGDFKDDLR.E, K.DAGGVMQGALGDFKDDLR.E	N/A
I_67*	179.9	46	5	5	Deamidation (NQ); Oxidation (M)	11923	R.AISESMANTGGGGLGGSR.A, R.AISESM(+15.99)ANTGGGGLGGSR.A	N/A
I_2074*	135.34	31	4	3	Amidation	9600	A.SYGPGPQASAAASR.L, Y.GPGPQASAAASR.L	Major ampullate spidroin 2 [Nephila senegalensis]
I_272*	122.08	33	4	3	Amidation	9371	Y.AAASQSAQVVSR.S, N.YAAASQSAQVVSR.S	Major ampullate spidroin 2 variant 1 [Nephila clavipes]
I_359*	188.07	51	28	28	Acetylation (Protein N-term); Deamidation (NQ); Ethylation; Dehydration; Dihydroxy	34638	A.GGLGGYGPGQQGPGQGGR.G, A.AAGGLGGYGP(+15.99)GQQGPGQQGPGQR.G	Major ampullate spidroin protein MaSp-h [Nephila clavipes]
I_1340	178.26	24	18	18	Carbamidomethylation; Deamidation (NQ); Ethylation; Dehydration; Dihydroxy; 4 more	49394	R.LSAPEAGTR.V, I.LSGP(+31.99)GR(+15.99)QASAAASR.L	Major ampullate spidroin protein MaSp-f isoform 1 [Nephila clavipes]
I_1327	147.26	40	12	12	Acetylation (Protein N-term); Deamidation (NQ); Ethylation; Dehydration; Hydroxylation; O-Diethylphosphorylation	14116	R.GQGGQGPSGQLAQAPSGYGQGSGAAAASGGLGGYGGQGGQR.S, G(+42.01)S(-18.01)GT(-18.01)AIAITAGGQR.G	Major ampullate spidroin 1 [Argiope trifasciata]
I_262	89.5	39	7	6	Acetylation (Protein N-term); Deamidation (NQ); Ethylation; Hydroxylation; O-Diethylphosphorylation	12276	G.Q(+28.03)GSGAAAAGAGQGGY(+15.99)GR.Q, G.AGAAAAAAGGAGQGGYGGLG.G	Dragline silk spidroin 1 [Nephila pilipes]
I_27560	129.55	71	5	2	Deamidation (NQ)	7995	R.SLGANSGEADAAGDR.G, G.AGAAAAAAGGAGQGGYGGLG.G	Dragline silk spidroin 1 [Nephila pilipes]
I_8908	111.49	30	3	3		9045	R.GYGPGSGAGAAAAGGAGEGGR.G, A.AAAAGGAGGEGGR.G	Minor ampullate spidroin protein MiSp-d [Nephila clavipes]
I_1660	34.34	1	3	3	Acetylation (Protein N-term)	438757	K.IALHLEQ.L, I.VATPDIAGV.H	N/A
I_9111*	33.72	44	3	3	Acetylation (Protein N-term); Deamidation (NQ)	11976	P(+42.01)GGYGPGQQGPGGYGPGQQ(+.98)GPGGAGAAAAAAAAGG.S, P(+42.01)GGYGPGQQGPGGYGPGQ(+.98)QGPGGAGAAAAAAAAGG.S	Major ampullate spidroin protein MaSp-h [Nephila clavipes]
I_3749	26.1	1	3	3	Deamidation (NQ)	269920	R.GGKRGN(+.98)THTKK.I, E.TLLSMN(+.98)PTR.G	N/A
I_23726	45.13	4	2	2		53637	L.LAADDFR.L, K.QNVKVRVASSSK.N	N/A
I_2904*	30.94	40	2	1		7155	G.GQGAGAAGAAAAAGGAGQGGYGGLG.G, G.QGGYGGLGGQGT.E	Dragline silk spidroin 1 [Nephila pilipes]
I_17778	28.19	31	2	1	Deamidation (NQ)	8397	G.YGGLGGQ(+.98)GTGAGGAAAA.G, S.ASLGGYGGLG.G	Major ampullate spidroin 1A precursor [Nephila clavipes]

Proteins from the corresponding transcriptome with 2 or more peptide matches were BLAST annotated (E-value cut-off 10^−3^). Example supporting peptides are shown (full list, see S4 Table). PTM, posttranslational modifications.

Beyond the matches made to the transcriptome, from the silk of *N*. *plumipes* and *N*. *pilipes* a further 2,420 and 2,658 *de novo* only peptides, respectively, were identified with high confidence (average local confidence above 70) that did not match any sequences within the transcriptome. This *de novo* dataset of unmatched potential proteins was BLASTed against NCBI protein databases, however the focus in this study is on those proteins relevant to their corresponding transcriptomes. Unlike a genome, a transcriptome can only provide us with genes transcribed at the time of RNA isolation and this may partly explain the discrepancy between matched and *de novo* peptides. A further explanation is that the hand-reeled silk also contains silk from different silk glands. Each silk gland ends at its own spigot on the surface of a spinneret. It is possible that as the silk thread passes past other spigots during collection, it also collects fibres from other silk glands. However, the spigots closest to the major ampullate spigot on the anterior lateral spinnerets produce pyriform spidroins and there was no evidence of these spidroins in the proteome [4]. Further, our gel-based extraction method might have missed proteins with relatively low molecular weight or low abundance, such as the cysteine-rich proteins identified by Pham et al. which were found to co-localise with spidroins in the MA silk of *Latrodectus hesperus* [8].

Quantitative analyses were undertaken and based on reads per kilobase of transcript per million mapped reads (RPKM) values by mapping the 2013 and 2016 data back to a combined *de novo* reference transcriptome. The 50 most highly expressed sequences of *N*. *pilipes* and *N*. *plumipes* were manually selected for further annotation (Tables 5 and 6). These abundant sequences were matched to sequences found in the NCBI or Uniprot public protein databases (accessed Oct-Dec 2017). Spidroins were, as expected, among the most highly expressed sequences of both datasets, numbering 23 and 26 spidroins for *N*. *plumipes* and *N*. *pilipes*, respectively. In both species, major and minor ampullate, and tubuliform spidroins were highly expressed in the MA gland. Interestingly, the *N*. *plumipes* sequence with the highest RPKM value could not be characterised based on BLAST protein prediction. Uncharacterised highly expressed sequences will be selected for functional annotation in future works.

**Table 5:** *Nephila plumipes* silk gland most abundantly expressed genes.

ID	RPKM	TPM	Match Description	E-value
U_3789	346459.71	282927.68	Uncharacterised protein [*Latrodectus hesperus*]	9.40E-05
U_18953	85599.66	62904.26	Major ampullate spidroin-like protein [*Nephilengys cruentata*]	1.50E-31
U_897	74973.89	55095.74	Cylindrical silk protein 1 [*Nephila clavata*]	4.40E-54
U_9403 *	55197.89	40563.04	Dragline silk fibroin [*Nephila clavipes*]	4.80E-29
U_999 †*	50336.66	36990.68	Major ampullate spidroin 1 [*Nephila clavipes*]	1.00E-33
U_20	49180.92	36141.37	N/A	
U_6066 *	46701.72	34319.50	Major ampullate spidroin protein MaSp-d [*Nephila clavipes*]	1.00E-37
U_10053	41232.70	33671.66	Major ampullate spidroin-like protein [*Nephilengys cruentata*]	4.70E-16
U_86	40804.39	29985.74	Tubuliform spidroin protein TuSp [*Nephila clavipes*]	4.70E-25
U_33	39225.86	32032.82	Major ampullate spidroin 1 [*Nephila clavipes*]	4.90E-49
U_440	32127.85	23609.65	Hypothetical protein NCL1_22245 [*Nephila clavipes*]	7.40E-38
U_10063 *	30452.03	22378.15	Major ampullate spidroin-like protein, partial [*Nephilengys cruentata*]	5.40E-32
U_56 †	27671.27	20334.67	Major ampullate spidroin 1 variant 2 [*Nephila clavipes*]	9.70E-102
U_137 †	24578.13	18061.63	Hypothetical protein NCL1_28494 [*Nephila clavipes*]	1.30E-69
U_172	22158.38	18095.09	Major ampullate spidroin 1A precursor, partial [*Nephila clavipes*]	9.30E-33
U_19886	22132.89	18074.27	Hypothetical protein NCL1_19751 [*Nephila clavipes*]	3.40E-08
U_63	22118.45	18062.48	N/A	
U_30462 †	19965.96	14672.30	N/A	
U_4890	18864.55	13862.91	N/A	
U_47	16143.09	13182.85	Dragline silk spidroin 1 [*Cyrtophora moluccensis*]	4.50E-18
U_11966	16061.76	11803.24	Major ampullate gland dragline silk protein-2, partial [*Araneus ventricosus*]	4.20E-17
U_22856 †	14927.17	12189.90	Uncharacterized protein LOC107452916 isoform X1 [*Parasteatoda tepidariorum*]	1.10E-12
U_339	13236.17	9726.81	N/A	
U_319	12939.36	9508.69	Spidroin protein Sp-907 [*Nephila clavipes*]	1.20E-56
U_17526	11596.29	8521.72	Major ampullate spidroin-like protein [*Nephilengys cruentata*]	4.10E-11
U_594	9427.11	7698.42	Tubulin alpha chain [*Stegodyphus mimosarum*]	0.00E+00
U_17052	9185.22	7500.88	Spidroin protein Sp-74867 [*Nephila clavipes*]	7.40E-43
U_14382 *	9074.23	6668.34	Major ampullate spidroin protein MaSp-d [*Nephila clavipes*]	2.40E-45
U_32 †	8925.67	7288.93	Hypothetical protein NCL1_19751 [*Nephila clavipes*]	1.30E-43
U_21571	8676.94	6376.38	Tubuliform spidroin-like protein [*Nephilengys cruentata*]	7.30E-42
U_16121	8344.15	6131.83	N/A	
U_6045 *	7092.78	5212.24	Spidroin 1 [*Nephila clavipes*]	3.00E-38
U_24501	6484.79	5295.65	Hypothetical protein NCL1_37350 [*Nephila clavipes*]	4.90E-13
U_460	6471.84	5285.06	Ferritin [*Stegodyphus mimosarum*]	2.60E-114
U_570	6287.70	4620.62	N/A	
U_82 †*	6223.69	4573.57	Major ampullate spidroin 1 variant 1, partial [*Nephila clavipes*]	9.20E-85
U_152	6087.77	4971.43	Elongation factor 1-alpha [*Stegodyphus mimosarum*]	0.00E+00
U_17648	5967.76	4385.50	N/A	
U_134 †	5059.94	4132.07	Cathepsin B [*Araneus ventricosus*]	0.00E+00
U_1088	4888.34	3592.28	Dragline silk protein spidroin 2 [*Nephila clavata*]	3.30E-45
U_346 †	4649.24	3416.57	N/A	
U_94	4571.75	3733.40	Nucleoside diphosphate kinase [*Latrodectus hesperus*]	3.50E-93
U_197 †	4458.19	3276.17	Putative fasciclin [*Latrodectus hesperus*]	9.40E-42
U_18	4188.49	3077.98	N/A	
U_8956 † *	4187.95	3077.58	Dragline silk fibroin [*Nephila clavipes*]	9.20E-07
U_30	4175.22	3068.23	Cytochrome c oxidase subunit I [*Cyclosa argenteoalba*]	0.00E+00
U_12055	4175.03	3068.08	N/A	
U_2628	4088.26	3004.33	Egg case protein variant 1 [*Argiope argentata*]	1.50E-38
U_318 †	4079.46	2997.85	N/A	
U_924 †	4009.24	2946.25	N/A	

E-value cut-off 10^−3^

† Genes found to exhibit a signal sequence

* Highly expressed genes also found within the silk proteome

**Table 6:** *Nephila pilipes* silk gland most abundantly expressed genes.

ID	RPKM	TPM	Match Description	E-value
I_7068	242027.26	162046.41	Major ampullate spidroin 2 variant 1 [*Nephila clavipes*]	7.70E-39
I_272 *	238666.86	159796.50	Major ampullate spidroin 2 variant 1 [*Nephila clavipes*]	7.70E-39
I_4212 †	221762.64	174740.14	Tubuliform spidroin 1 [*Araneus ventricosus*]	5.20E-11
I_2074 *	138074.73	92446.26	Major ampullate spidroin 2 [*Nephila senegalensis*]	5.00E-13
I_8400	77285.64	60898.01	Tubuliform spidroin 1 [*Agelenopsis aperta*]	5.90E-18
I_16679	60136.69	40263.79	Major ampullate spidroin 2 [*Nephila clavipes*]	3.40E-35
I_9111 *	53644.90	35917.29	Major ampullate spidroin protein MaSp-h [*Nephila clavipes*]	1.30E-59
I_33885 †	44532.47	29816.18	Dragline silk fibroin [*Araneus ventricosus*]	1.40E-20
I_33 *	44168.19	34802.78	NA	
I_52	34336.68	22989.71	Spider venom protein NPTX_B154 [*Nephila pilipes*]	3.20E-10
I_5 †	33908.87	26718.84	NA	
I_359 *	32815.12	21970.96	Major ampullate spidroin protein MaSp-h [*Nephila clavipes*]	1.60E-136
I_67 *	26171.69	20622.25	NA	
I_820	25576.95	17124.73	Dragline silk spidroin 1 [*Nephila pilipes*]	1.80E-94
I_1332 †	23250.68	15567.21	Spider venom protein NPTX_C786 [*Nephila pilipes*]	1.50E-12
I_3479 †	21411.41	14335.75	Hypothetical protein NCL1_21799 [*Nephila clavipes*]	3.00E-04
I_9940	20210.87	15925.36	Cylindrical silk protein 1 [*Nephila clavata*]	4.90E-12
I_2337 †	20187.99	13516.63	NA	
I_77	19281.52	12909.71	ART2 [*Enterospora canceri*]	4.40E-57
I_550	18520.57	12400.23	Major ampullate spidroin protein MaSp-h [*Nephila clavipes*]	1.20E-58
I_12595	17160.99	11489.93	Dragline silk fibroin, partial [*Araneus ventricosus*]	1.80E-42
I_79	15849.11	12488.47	Hypothetical protein NCL1_39416 [*Nephila clavipes*]	2.10E-14
I_178 *	15167.42	11951.32	NA	
I_16079	15154.62	10146.59	NA	
I_10698	15064.25	11870.03	Minor ampullate spidroin-like protein [*Nephilengys cruentata*]	4.10E-63
I_5861 †	14879.35	9962.29	Hypothetical protein NCL1_19751 [*Nephila clavipes*]	3.40E-39
I_22088	14056.52	11075.98	Tubuliform spidroin 1 variant 1 [*Araneus diadematus*]	6.70E-07
I_9	13762.70	10844.46	Minor ampullate fibroin 1 [*Nephila antipodiana*]	1.90E-45
I_12551 †	13639.82	10747.64	Hypothetical protein NCL1_19751 [*Nephila clavipes*]	3.60E-31
I_3787	13233.55	10427.51	Minor ampullate silk protein MiSp1 [*Nephila clavipes*]	8.00E-27
I_576	13206.17	10405.94	Bm3878 [*Brugia malayi*]	4.50E-40
I_37 †	12356.47	9736.41	Venom allergen 5 [*Stegodyphus mimosarum*]	1.10E-41
I_7754	11827.42	9319.54	Minor ampullate spidroin protein MiSp-a [*Nephila clavipes*]	6.00E-16
I_60663	11758.95	9265.58	NA	
I_2057 †	10260.75	6869.96	Hypothetical protein NCL1_37703 [*Nephila clavipes*]	8.10E-15
I_4156 *	10236.26	6853.56	Minor ampullate spidroin [*Argiope argentata*]	7.10E-27
I_2304	10148.69	6794.93	Hypothetical protein THAOC_21441 [*Thalassiosira oceanica*]	2.50E-22
I_20587	9866.14	7774.13	Minor ampullate spidroin-like protein [*Nephilengys cruentata*]	2.00E-56
I_8429	9848.94	7760.57	NA	
I_7810 *	9654.94	7607.71	UniProt BLAST Minor ampullate silk protein MiSp1 [*Nephila clavipes*]	4.50E-39
I_2904 *	9607.91	6432.86	Dragline silk spidroin 1 [*Nephila pilipes*]	6.00E-33
I_31392	9573.32	7543.40	CRISP/Allergen/PR-1 [*Parasteatoda tepidariorum*]	6.40E-07
I_55 †	9455.94	7450.90	Cylindrical silk protein 1 [*Nephila clavata*]	6.40E-60
I_55059	9033.46	6048.25	NA	
I_23940	8958.45	7058.90	Cylindrical silk protein 1 [*Nephila clavata*]	1.80E-66
I_251	8938.21	5984.47	Putative tumor differentially expressed protein [*Latrodectus hesperus*]	2.10E-21
I_4316	8829.59	6957.37	Minor ampullate spidroin-like protein [*Nephilengys cruentata*]	1.70E-30
I_710	8760.58	5865.54	NA	
I_7 †	8494.88	6693.63	Hypothetical protein X975_26006 [*Stegodyphus mimosarum*]	1.90E-05
I_26363	8262.29	6510.35	Minor ampullate spidroin-like protein [*Nephilengys cruentata*]	1.40E-57

E-value cut-off 10–3

† Genes found to exhibit a signal sequence

* Highly expressed genes also found within the silk proteome

This study found the MA gland alone produces six of the seven classes of silk products: MA, minor ampullate, flagelliform, tubuliform (also at times referred to as cylindriform silk), aciniform and aggregate silk products. Several other studies have also found multiple spidroin types expressed in a single gland [6, 7, 49, 50]. The only silk product not found to be produced by the MA gland of both *N*. *pilipes* and *N*. *plumipes* was pyriform adhesive silk, which is used to attach threads to objects and to each other [51]. The processing duct of the pyriform gland is shorter than most other ducts suggesting other silks require more extensive processing, which may explain why this silk is absent from the MA gland transcriptome. However, pyriform products are the least intensively studied of the spider silk repertoire, and the lack of pyriform annotation in our MA databases may be a reflection of poor representation in the public databases at the present time [51, 52].

Interestingly, in both *N*. *plumipes* and *N*. *pilipes*, tubuliform spidroins were found to be more highly expressed in the 2016 MA gland transcriptomes yet not expressed in the 2013 transcriptomes (see Table 1 and 2). Tubuliform silk is produced during reproduction for the formation of egg sacs [4, 53]. While no spiders were gravid at the time of dissection, it is possible they were collected and dissected just after the production of an egg sac, or just prior to vitellogenesis, and the 2016 transcriptomes reflect this in their relatively high expression of tubuliform silk transcripts. Expression of tubuliform spidroins in the MA gland has been previously noted in transcriptomic studies [50]. Vasanthavada et al. suggest that spiders can downregulate the production of various silks to maintain MA spidroin synthesis as an energetic trade-off, and Larracas et al. suggest that female spiders may shift synthesis of MA gland spidroins to tubuliform spidroins during the reproductive stage [6, 54, 55]. Our study did not find tubuliform spidroins in the silk proteome, however, the silk was collected and digested at the same time as the 2013 transcriptomes. It would be interesting to see if tubuliform spidroins could be found within the dragline silk of spiders prior to, during, or just post egg sac production.

This study is the first silk gland-specific transcriptome and proteome analysis in these Australian golden orb-weaving species. Major ampullate transcriptome analysis procured sequences for all silk types thus far known for golden orb spiders with the exception of pyriform adhesive silk. We found differential expression of tubuliform silk in the MA gland, suggesting a greater role for this gland producing tubuliform silks during spider reproduction. The silk proteome analysis resulted in 29 and 18 proteins for *N*. *plumipes* and *N*. *pilipes* that match to their corresponding MA gland transcriptomes.

## Supporting information

S1 Table *Nephila plumipes* proteins with homology to *Nephila clavipes* spidroins. (XLSX)

S2 Table *Nephila pilipes* proteins with homology to *Nephila clavipes* spidroins. (XLSX)

S3 Table List of peptides that were mapped back to the *Nephila plumipes* transcriptome. (XLSX)

S4 Table List of peptides that were mapped back to the *Nephila pilipes* transcriptome. (XLSX)
